# Clinical Translational Potentials of Stem Cell-Derived Extracellular Vesicles in Type 1 Diabetes

**DOI:** 10.3389/fendo.2021.682145

**Published:** 2022-01-12

**Authors:** Wei Hu, Xiang Song, Haibo Yu, Jingyu Sun, Hongjun Wang, Yong Zhao

**Affiliations:** ^1^ Center for Discovery and Innovation, Hackensack Meridian Health, Nutley, NJ, United States; ^2^ Department of Chemistry and Chemistry Biology, Stevens Institute of Technology, Hoboken, NJ, United States; ^3^ Throne Biotechnologies Inc., Paramus, NJ, United States

**Keywords:** extracellular vesicle, stem cell, type 1 diabetes, exosome, autoimmunity, immunomodulation, β-cell regeneration

## Abstract

Type 1 diabetes (T1D) is an organ-specific disease characterized by the deficiency of insulin caused by the autoimmune destruction of pancreatic islet β cells. Stem cell-based therapies play essential roles in immunomodulation and tissue regeneration, both of which hold great promise for treating many autoimmune dysfunctions. However, their clinical translational potential has been limited by ethical issues and cell transplant rejections. Exosomes are small extracellular vesicles (EVs) released by almost all types of cells, performing a variety of cell functions through the delivery of their molecular contents such as proteins, DNAs, and RNAs. Increasing evidence suggests that stem cell-derived EVs exhibit similar functions as their parent cells, which may represent novel therapeutic agents for the treatment of autoimmune diseases including T1D. In this review, we summarize the current research progresses of stem cell-derived EVs for the treatment of T1D.

## Introduction

Type 1 diabetes (T1D) is an autoimmune disorder characterized by impaired blood sugar control and insulin deficiency due to an autoimmune destruction of insulin-producing cells in the pancreas (1). Long-term hyperglycemia increases the risks of developing a number of diabetes-associated complications such as cardiovascular disease, kidney diseases, stroke, and blindness. These complications lead to a significant reduction in the quality of life for those affected (2). While the exact pathogenesis of T1D remains unknown, it is associated with a combination of environmental factors and genetic predisposition (3). The administration of exogenous insulin only alleviates symptoms and cannot fully mimic the physiological actions of endogenous insulin released from healthy pancreata. Pancreatic islet transplantation has become a potential treatment for T1D; however, drawbacks impeding its widespread application include high costs, a shortage of islet donations, and lifelong utilizations of immune-suppression drugs post-transplantation (4, 5). Recently, functional insulin-producing cells have been generated from embryonic stem cells (ESC) and induced pluripotent stem cells (iPSCs) (6–8). This has led to clinical trials for the treatment of T1D subjects including ViaCyte studies with VC-01 and VC-02 products (NCT04678557 and NCT03163511, respectively) and a Vertex study with VX-880 (NCT04786262). Clinical applications of stem cell-derived insulin-producing cells may have ethical and safety concerns including potential tumor formations and immunological reactions (9). Therefore, understanding how to correct autoimmunity and overcome the deficiency of islet β cells are two critical concerns for the treatment of T1D.

Extracellular vesicles (EVs) are groups of small membranous particles that are released by various cells and play an essential role in the transfer of information between adjacent and distant cells (10), as well as the mediation of numerous physiological and pathophysiological processes (11). Since EVs have been found in all mammal biofluids, EVs containing biomolecules (RNAs and proteins) have been widely applied as biomarkers for diagnosis of diseases (12). Notably, increasing evidence has demonstrated the therapeutic potentials of EVs in various diseases including cancer, autoimmune diseases, and infection diseases (13, 14). To date, increasing evidence has demonstrated that stem cell-derived EVs with similar functions as their parent cells not only contribute to the promotion of tissue regenerations (15) but also the modulation of various functions of immune cells (16) and amelioration of autoimmunity in islets (17, 18). This review will focus on current advancements of stem cell-derived EVs for the treatment of T1D with a special focus on their immune modulations and therapeutic potentials to overcome the deficit of islet β cells.

## Extracellular Vesicles

### EV Biogenesis and Isolation

EVs are divided into three categories according to their subcellular origin and secretion mechanisms: exosomes (30–150 nm), microvesicles (MVs, 100–1,000 nm), and apoptotic bodies (1,000–5,000 nm) (19). MVs, also termed “microparticles”, are generated through direct budding at the plasma membrane. Apoptotic bodies are relatively large particles, with sizes ranging from 500 to 2,000 nm in diameter and derived from the late stage of apoptotic cells (19, 20). Exosomes, the smallest vesicles, are derived from endosomal budding and released into the lumen through exocytosis (21, 22). To explore the physiological and therapeutic functions of exosomes, the purification and quantification of exosomes are necessary to meet the requests of basic science and clinical practice. Several methods have been utilized to facilitate the isolation of EVs including precipitations, immune-affinity capture, ultracentrifugation (UC), sucrose density gradient ultracentrifugation, and size exclusion chromatography (SEC) (23). Each method is based on one particular feature of EVs, such as density, size, and surface-specific proteins. These methods have certain limitations in the purity and low yield of exosomes (24). Among these techniques, ultracentrifugation is the “gold” standard and is widely accepted for EVs experimental research (25). Recently, the microfluidics-based method has advanced exosome isolation with the high purity and high yield of exosomes (26). In the field of EVs, most studies have focus on the exosomes. Due to the overlapping range of size and density, as well as lack of specific protein markers for these three subtype EVs, the purification of separated exosomes, MVs, and apoptotic bodies is technically complicated. In this review, we use the term “EVs” on behalf of exosomes and MVs, and apoptotic bodies.

### Composition of EVs

The membrane of EVs consisting of lipid bilayer is similar to that of cell plasma membrane and is in contrast with the single-layered high-density lipoprotein (HDL) and low-density lipoprotein (LDL) found in body fluids (27). Molecular characteristics revealed that there are a variety of biomolecules such as RNAs, DNA, proteins, and lipids inside EVs. Exosomes from different sources contain certain source-specific molecules, as well as common molecules found across all types of exosomes. Exosomes manufactured from the endocytic pathway inherit endosomal components such as Alix and Tsg101 molecules. Other molecules, including tetraspanin (CD9, CD63, and CD81), membrane molecules (integrins), and intercellular adhesion molecule 1 (ICAM-1), and cytoskeletal components (tubulin, actin, and annexins) are universally presented in almost all types of exosomes derived from different sources of cellular (28, 29). Microvesicle cargo was dependent on the cellular source since the formation of MVs was directly generated from outward budding of cell plasma membrane, along with cytosolic and plasma membrane-associated proteins (30). Several proteins commonly identified in MVs include tetraspanins, cytoskeletal proteins, heat shock proteins, and integrins (31). However, there were no EV-specific markers to distinguish MVs from exosomes. The compositions of apoptotic bodies were different among exosomes and MVs, in that they contain the degraded protein, DNA fragments, or even intact organelles (32). Therefore, recent studies showed that native EVs carry biological cargo from different cells acting as novel mediators. They may contribute to intercellular communications and modulate the recipient cells’ function and demonstrate how EVs serve as biomarkers and therapeutic agents for both diagnosis and treatment among various diseases (33, 34).

## Therapeutic Potentials of Stem Cell-Derived Exosomes for the Correction of Multiple Immune Dysfunctions in T1D

**Figure 1:**
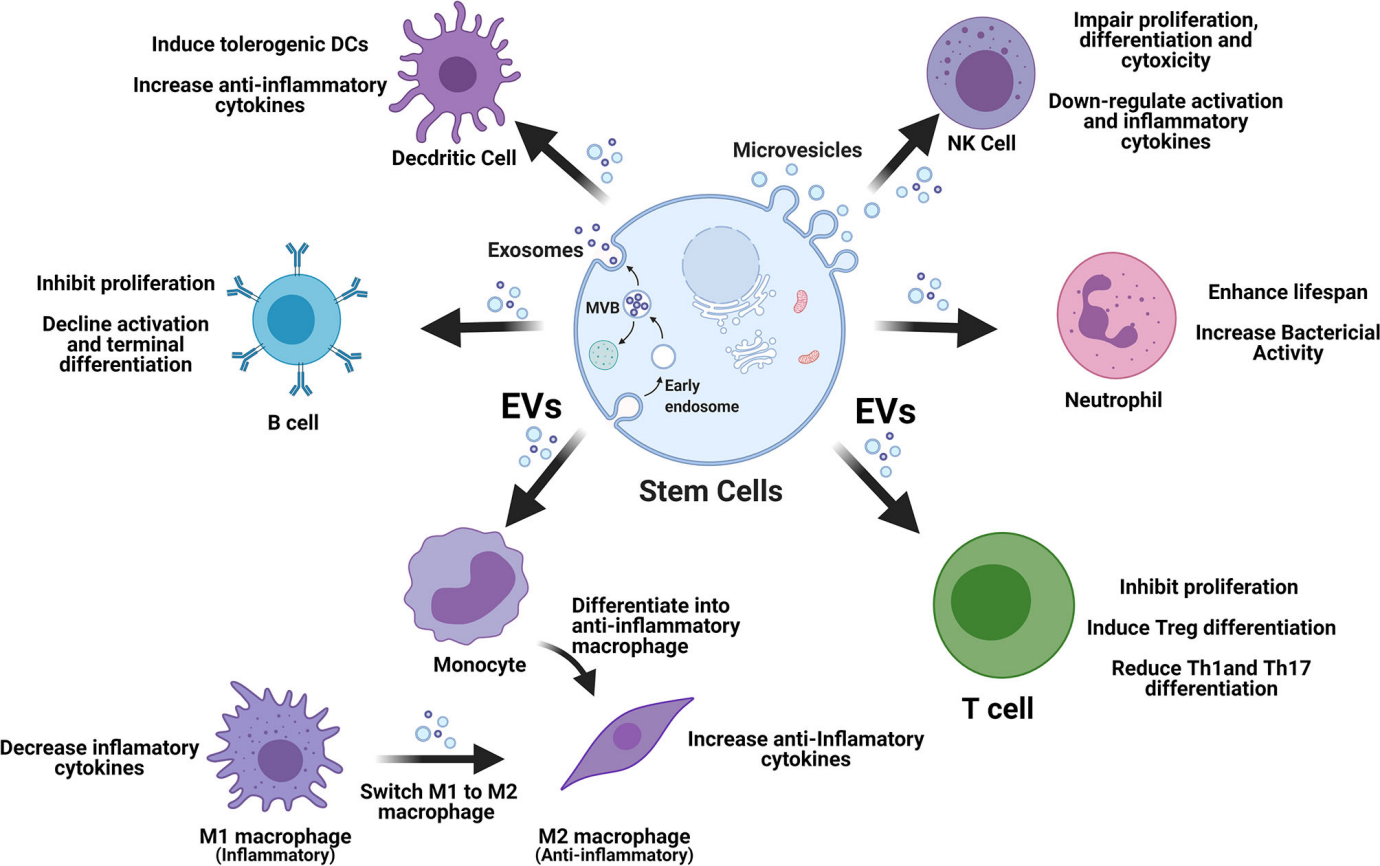
Stem cell-derived EVs display multiple immune regulations on different types of immune cells. EVs, extracellular vesicles; M1, type 1 macrophages; M2, type 2 macrophages; NK, natural killer cells; Tregs, regulatory T cells.

Stem cell-derived EVs display great potential in immune modulation, which may be translated into clinical treatment of T1D. The released EVs enter into circulation and target different cells *via* direct fusion with plasma membrane, endocytosis by phagocytosis, or receptor–ligand interaction (35). Immediately, after EV’s molecular content (miRNAs and proteins) is released into these targeted cells, contributing to the immunomodulation through different signaling pathways (36, 37). In the following section, we review current advancements for the modulation of stem cell-derived EVs on different immune cell compartments as alternative approach to correct the immune dysfunctions in T1D (**Figure 1**).

### Immune Modulation of Stem Cell-Derived EVs on T Cells

T cells as pathogenic effector cells in type 1 diabetes are well established. Both CD4+ and CD8+ cells play distinct and highly pathogenic roles driving diabetogenesis. CD8+ T cells are the predominant and inflammatory T-cell population contributing to the destruction of pancreatic islet β cells. Wiedeman et al. found that the rapid loss of C-peptides in T1D subjects is associated with prevalent islet-specific CD8+ memory T cells (38). CD8+ T cells predominantly infiltrate the islets, yet their activations are primed by CD4+ T cells (39). For example, diabetogenic CD8+ T-cell functions are maintained by CD4+ T cells in diabetic pancreata (40). CD4+ T cells can give rise to different functional subsets in response to different signals, offering to “help” effector immune cells in their immune response (41). Increasing evidence has demonstrated that multiple CD4+ T cells are involved in the development of insulitis, including T-helpers type 1, 2, and 17 (Th1, Th2, and Th17), regulatory T cells (Treg), and follicular B helper T cells (Tfh) (42–44). Studies have revealed an altered balance between Th1/Th17 and Th2 immune responses leading to T1D (45, 46). Tregs are critical regulators of peripheral tolerance, with defects in Treg phenotypes and suppressive capacities being reported in T1D patients (47–49). Tfh cells regulate germinal center (GC) formation and humoral response (50). Recently, studies have found that long-lived plasma cells secrete T1D-associated autoantibodies generated from GCs with the help of Tfh cells (51).

Stem cells with strong immune modulations have been applied to correct the dysfunction of T cells in T1D diabetes. Researchers found that both CD4+ and CD8+ regulatory T cells increase after coculturing with MSC. Fiorina et al. claim that allogeneic MSC administration can shift the Th1/Th2 balance among T1D mice models (52). Furthermore, several studies have reported that MSC could induce Treg differentiation and restore the balance between Th1/Th17s and Tregs through *ex vivo* and animal tests (53, 54). Moreover, cord blood-derived stem cells (CB-SC), with the effective immune modulation function, have been applied in the Stem Cell Educator^®^ therapy for the treatment of T1D and autoimmune diseases. The attached CB-SC coculture with patient’s apheresis mononuclear cells (MNC) for a short period (8–17 h). Consequently, the CB-S-educated MNC cells are returned back into the patient’s blood circulation through infusion (55). Our previous clinical studies have demonstrated that Stem Cell Educator therapy utilizing cord blood-derived stem cells (CB-SC) can increase Th2-related cytokines (IL-4 and IL-12), decrease the level of Th17-associated cytokine (IL-17) (55), and reduce the percentages of CD4+ and CD8+ effector memory T cells (T_EM_) in T1D patients (56). Additionally, mechanistic studies have demonstrated the direct downregulation of the anti-CD3/CD28 bead-activated CD4+HLA-DR+ and CD8+HLA-DR+ T cells after the treatment with CB-SC-derived exosomes (57).

Stem cell-derived EVs display similar characterization as their parent cells and play an essential role in modulations of the T-cell proliferation, differentiation, and apoptosis (58, 59). It has been reported that stem cell-derived EVs inhibit the proliferation of T cells through the delivery of their specific protein or miRNAs (60). Stem cell-derived EVs could increase the percentage of Tregs and decrease Th1/Th17 subpopulations (61), highlighting the therapeutic potential of stem cell-derived EVs for the treatment of T1D. Recently, Shigemoto-Kuroda et al. found that human MSC-derived EVs can effectively prevent the onset of T1D in animal models. Using the mixed lymphocyte reaction (MLR) assays, these MSC-EVs are able to suppress the proliferation of Th1 and Th17 cells, which play a key role in the prevention of T1D onset (62). Similarly, Favaro et al. found that MSC-derived EVs can decrease the number of Th17 cells and cytokine IL-17, as well as increase the percentage of Tregs in peripheral blood mononuclear cells (PBMC) from recent-onset T1D patients (63). Additionally, this group found that MSC-EVs could decrease the level of IFN-γ in GAD65-stimulated PBMC and increase the level of anti-inflammation molecules, including the transforming growth factor-β (TGF-β), IL-10, and prostaglandin E2 (PGE-2) (64). Stem cell-derived EVs therefore have significant importance for T1D therapy. Nojedehi et al. applied EVs from adipose tissue-derived MSC (50 μg/ml in PBS, i.p., twice/week/mouse) for the treatment of chemical streptozotocin (STZ)-induced diabetic mice. Their study found that MSC-EVs are helpful in maintaining blood glucose and body weight. What is more, a mechanistic study revealed that there were significant upregulations in the levels of IL-4, IL-10, and TGF-β, as well as markedly decreased levels of IFN-γ and IL-17 among T1D mice after the treatment with MSC-derived EVs (65). Collectively, these data indicate the therapeutic potentials of stem cell-derived EVs as a novel approach to treating T1D through their immune modulations on T cells.

### Immune Modulation of Stem Cell-derived EVs on the Antigen-Presenting Cells

Macrophages, one of the major antigen-presenting cells, have vital roles in the innate immune responses, glucose and lipid metabolism, and pathogenesis of diabetes (66, 67). Macrophages can be simplified into two subsets—pro-inflammatory (M1) and anti-inflammatory (M2) profiles (68). M1 macrophages are induced by Th1-related cytokines (IFN-γ and TNF-α) or microbial products [Toll-like receptors (TLRs), ligands, and lipopolysaccharide (LPS)] to kill pathogens and present their antigens to T cells for adaptive responses. M1 macrophages exhibit high levels of phagocytic activity and enhanced antigen-presenting capability through the expression of M1-associated markers including CD80, CD86, and nitric oxide synthase (iNOS). Additionally, M1 macrophages produce high levels of proinflammatory cytokines such as interleukin-12 (IL-12), IL-1β, and IL-23. In contrast, M2 macrophages are induced by Th2-response cytokines (IL-4 and IL-13). M2 macrophages are characterized by an anti-inflammatory profile that plays a crucial role in permitting the resolution of tissue repair (69, 70) and secrete a variety of anti-inflammatory mediators (e.g., IL-10 and TGF-β1) and reduce the level of proinflammatory cytokines secreted by M1 macrophages (71). In addition, M2-associated markers include CD163, mannose receptor (CD206), STAT6, and arginase 1 (72, 73). M2 macrophages with high expressions of arginase result in the production of polyamines and collagen, both of which favor tissue remodeling (74). Moreover, M2 macrophages with increased activity of arginase-1 can lower the level of NO secretion by competing for L-arginine, the substrate of iNOS (75). Both macrophages are essential players in the pathogenesis of T1D. While M1 macrophages trigger the immune response and initiate insulitis, M2 macrophages act as negative regulators by decreasing inflammation and insulitis in the T1D pancreas (70).

Macrophages may directly provoke or enhance insulin secretion through the production of factors such as retinoic acid (76). The depletion of islet-resident macrophages limits the islet leukocytic infiltration during early phases of diabetogenesis (77, 78). Both macrophage populations are central players in diabetes. M1 macrophages are responsible for triggering the inflammatory response, initiating insulitis and pancreatic cell death during the onset of T1D. M2 macrophages decrease hyperglycemia, insulitis, and inflammation in the pancreas, thereby negatively regulating T1D development (70). Fernando et al. found that LPS re-stimulation in diabetic bone marrow-derived macrophages (BMDM) resulted in higher secretions of TNF-α compared to non-diabetic BMDM (79). What is more, their study found that long-term high glucose-treated macrophages increased the levels of inflammatory cytokines (e.g., IL-1β and TNF-α) in macrophages (80). Similarly, Ferris et al. reported that an increased inflammatory signature in islet macrophages of non-obese diabetic (NOD) mice was correlated with the elevated expressions of chemokines and oxidative responses (81).

Increasing evidence has demonstrated that stem cell-derived EVs can reduce the inflammation through targeting macrophages (82, 83). Stem cell-derived EVs can both promote M2 macrophages and suppress M1 macrophage polarization by upregulating anti-inflammation cytokines and downregulating inflammation-related cytokines (84, 85). Interestingly, researchers have reported that MSC exosome-treated macrophages can reduce the inflammation and T-cell proliferation (86, 87). Recently, our studies demonstrated that CB-SC-derived exosomes can favorably target monocytes in the presence of PBMC and polarize these monocytes into M2 macrophages (57, 88). These functions may contribute to the clinical therapeutic potentials of Stem Cell Educator therapy to treat T1D (55) and other inflammatory-associated diseases (89).

Dendritic cells (DC) are another major population of antigen-presenting cells. Recent studies suggest that diabetic subjects have impaired functions of DC that may contribute to the pathogenesis of T1D (90, 91). Their findings revealed that adenosine deaminase (ADA) is upregulated in NOD dendritic cells, which induce their spontaneous activation. Therefore, transferring the ADA-deficient DC to NOD mice can protect them from the development of diabetes (92). MSCs have been shown to inhibit the maturation and function of bone marrow-derived DC (BMDC) and induce the differentiation of DC into tolerogenic dendritic cells (93, 94). Similarly, MSC-derived exosomes suppress the maturation of BMDC with decreased secretion of pro-inflammatory cytokines (IL-12) and increase the production of anti-inflammatory cytokines (TGF-β), contributing to the DC-induced immune responses (95, 96). Mechanistic studies have demonstrated that MSC-derived exosomal miRNA-146a play an essential role in the immunomodulatory function of DC (97). Furthermore, Favaro et al. reported that the treatment of DC with MSC-derived EVs can increase the percentage of Tregs and decrease Th17, thus potentially leading to the inhibition of inflammatory T-cell response to islet antigens (63).

### Immune Modulation of Stem Cell-Derived EVs in Other Immune Cells

In addition to T cells and monocytes/macrophages involved in the initiation of T1D, other immune cells contribute to the development of T1D (98, 99). Natural killer (NK) cells may be involved in the pathophysiology of diabetes since they partner with antigen-presenting cells or T cells for killing the targeted islet β cells (100). Literature has demonstrated that NK cells also release cytokines that transmit adaptive immunity. *In vitro* studies have confirmed that NK cells can lyse islet cells (101). Animal studies have demonstrated that the depletion of NK cells can significantly decrease T1D development (102). Stem cells can interact with NK cells for modulating these NK functions (103). For example, MSCs can inhibit IL-2-inuduced NK cell proliferation and downregulate expressions of activating NK receptors (104). Recently, MSC-derived exosomes were shown to reduce the release of interferon gamma (IFN-γ) and tumor necrosis factor alpha (TNF-α) by activated NK cells, alleviating the inflammatory response (105). Moreover, Fan and colleagues reported that human fetal liver MSC-derived exosomes impair NK proliferation, differentiation, and cytotoxicity through exosome-associated TGF-β (106).

B cells have an important role in the adaptive immune response including antibody production, antigen presenting, and multiple cytokine production (107, 108). Although there is much evidence that T cells play a major pathogenic role in T1D, B cells are also required for the development of diabetes, which has been demonstrated by the depletion of B cells using anti-CD20 or anti-CD22 monoclonal antibodies (mAb) (109, 110). Additionally, depleting B cells with anti-CD20 mAb in patients with newly diagnosed T1D can preserve islet β-cell function and delay the requirement for insulin administration among 1-year follow-ups (111, 112). Stem cells have proven immunomodulatory properties for both the activation and proliferation of B cells as well as the induction of regulatory B-cell generation (113, 114). MSC-derived EVs can affect the mRNA expression of B cells and impair their proliferation (115). Adamo et al. reported that MSC-derived exosomes can inhibit the proliferation and activation of B cells by downregulating their PI3K-AKT signaling pathway through the delivery of exosomal miR-155-5p (116).

Neutrophils are the primary innate cells to be recruited to the sites of inflammation, as they provide the front line of defense against the infection. Neutrophil functions have been reported to be closely related to β‐cell autoimmunity, as a significant decrease in neutrophil numbers and chemotactic activity can be detected in T1D patients, but not among the healthy controls (117, 118). Additionally, the impaired phagocytosis and bactericidal activity of neutrophils were shown in marked correlation with the elevated blood glucose levels (119). There is more evidence demonstrating that treatment with stem cells can enhance the lifespan and bactericidal activity of neutrophils (120–122). Interestingly, since EVs have similar functioning as their parent cells, stem cell-derived EVs could significantly prolong the survival and function of neutrophils (123, 124).

## Therapeutic Potentials of Stem Cell-Derived EVs for Overcoming the Shortage of Islet Beta Cells in T1D

Pancreatic β cells are the only specialized cells in mammals that can secrete insulin. Cytoarchitectural studies in rodents have shown that they are located in the core of the rodent pancreatic islets and are surrounded by α cells, which can secrete glucagon, δ cells, which are few in number and secrete somatostatin, and PP cells, which secrete pancreatic polypeptide (125). Conversely, human islet β cells, α cells, and δ cells were found scattered through the human islets (126). The destruction of pancreatic β cells results in absolute insulin secretion deficiency—the hallmark characteristic of T1D (127). Understanding how to restore the islet β-cell population is one of the most challenging fields in the treatment of T1D. Nevertheless, a number of hurdles must be overcome. Currently, several known approaches have been applied to β-cell regeneration including endogenous regeneration of β cells, *in vitro* β-cell regeneration using stem cells, and the promotion of the remaining β cells that have survived (128, 129). Stem cells have great differentiation potential, being able to differentiate into endogenous β cells or *in vitro* insulin-producing cells. In addition, stem cells have powerful modulation functions for promoting the survival of remaining β cells (130, 131). Recently, more evidence has suggested that stem cell-derived EVs have positive effects for promoting the survival of β cells and generation of insulin-producing cells (132, 133).

### Effects of Stem Cell-Derived EVs on Endogenous β-Cell Proliferation and Transdifferentiation

Endogenous regeneration of β cells, which occurs through the stimulation of existing β-cell proliferation or differentiation of other pancreatic progenitor cells or stem cells into functional insulin-secreting cells (designated neogenesis), is a potential strategy for residual β-cell replication and neogenesis to treat diabetes (134). Pancreatic β-cell replication occurs readily during the fetal and neonatal stages and then declines after these stages. Increasing evidence has demonstrated that several mitogenic signaling pathways mediate the replication of β cells including IRS-PI3K-AKT, GSK3,cMYC, RAS/RAF/ERK, and mTOR (135). Interestingly, insulin signaling can regulate the mitotic FoxM1/PLK1/CENP-A pathway for promoting adaptive β-cell proliferation (136). In addition, multiple soluble factors, including GLP-1, lepin, and IL-6, have been implicated to control the proliferation of β cells. For example, studies have demonstrated that the transforming growth factor (TGF)-beta family and WNT/beta-Catenin signaling serve as the potential keys to controlling β-cell proliferation and differentiation (137–139). Moreover, increasing evidence has demonstrated that stem cell infusions can enhance the cell proliferation during the process of tissue repair (140, 141). Recently, scientists have applied human MSC with overexpressions of telomerase reverse transcriptase (TERT) to enhance the proliferation of autochthonous pancreatic β cells in half-pancreatectomized mice (142). Also, stem cell-derived EVs cause positive effects for the treatment of T1D by promoting existing β-cell proliferation. For instance, Mahdipour et al. found that applying EVs from menstrual blood-derived MSCs (10 μg/rat, i.v., on day 0, 2, and 10 after the injection of streptozotocin, respectively) can restore the β-cell mass and insulin production in diabetic rats. Mechanistic studies have revealed that EVs can induce β-cell regeneration through the activation of the pancreatic and duodenal homeobox 1 (PDX-1) pathways (143). Additionally, the reprogramming of cells into β-like cells has drawn increasing attention among the research community as an alternative means for endogenous β-cell regeneration. According to the development of embryonic pancreatic β cells, transcription factors play an essential role in pancreatic β-cell determination including PDX1, NGN 3, SOX9, NKX6.1, MAFA, and MNX1 (144). For example, Zhou et al. reported that the re-expression of transcription factor NGN 3, combined with PDX1 and MAFA, can efficiently reprogram pancreatic exocrine cells into insulin-producing cells (145). Additionally, their study found that the overexpression of Pref-1-activated MAPK and AKT signaling can help to increase insulin synthesis *via* the differentiation of human pancreatic ductal cells into β-like cells (146). Recently, research has demonstrated that by disrupting α cell-specific TFs such as DNMT1 and ARX, the reprogramming of α cells to β cells can be achieved (147). Until now, there has been a lack of direct evidence demonstrating that stem cells and their derived EVs can modify the process of reprogramming non-pancreatic cell into insulin-expression cells. However, Ribeiro et al. found that human pancreatic islet-derived EVs improve the differentiation of iPSC cluster culture in 3-D collagen hydrogel with increased pancreatic marker expression (148). Recently, Oh and colleagues showed that β-cell-derived EVs can directly trigger the differentiation of bone marrow mononucleated cells into insulin-producing cells (149). These findings suggest that stem cell-derived EVs may serve as possible mediators for the development of insulin-producing cells from non-beta pancreatic cells. Prospectively, investigating the function of stem cell-derived EVs on endogenous regeneration of β cells will provide deep insights into the process of cell reprogramming.

### Protective Effects of Stem Cell-Derived EVs on Islet β Cells and Pancreatic Islets

The promotion of β-cell survival and functioning can be achieved *via* apoptosis protection. The apoptotic pathway mainly consists of two pathways—the extrinsic and intrinsic pathways. The extrinsic pathway is ignited by the cell surface death receptor (Fas and tumor necrosis factor receptor) bound with their ligand. The intrinsic pathway, also termed the “mitochondrial-mediated pathway”, is triggered by the pre-apoptotic Bcl-2 family, leading to permeabilization of the mitochondrial outer membrane. Both pathways culminate in the activation of the caspase protease family, ultimately resulting in the dismantling of cells (150, 151). Autophagy is a cell survival mechanism that delays the cell death. There are increasing evidence supporting that coculture stem cells with β cells can delay the apoptosis of β cells (152, 153). Mechanistic studies have found that the secretome from stem cells can enhance autophagy and exert the protective effects on β cells (154, 155). Recently, researchers found that EVs among MSC secretome play a powerful role in the anti-apoptosis of β cells. Keshtkar et al. demonstrated that MSC-derived EVs can improve islet survival and function by upregulating insulin and vascular endothelial growth factor (VEGF) expressions (156). Furthermore, mechanistic studies have revealed that MSC-derived EVs preserve β-cell function, depending on their contained miRNA-21 for alleviating ER stress, and downregulate p38 MAPK phosphorylation to reduce hypoxia-induced apoptosis of β cells (157). Furthermore, *in vivo* studies have affirmed these findings, demonstrating that the administration of EVs from MSC can restore insulin secretions by inhibiting STZ-induced β-cell apoptosis in T1D mouse models (158).

Additionally, stem cell-derived EVs have a strong ability to promote angiogenesis during the tissue repair. An *in vitro* study found that human bone marrow stem cells enhance islet vascularization and preserve islet function with significantly increased expressions of insulin (159). Moreover, administration of MSC-derived EVs can preserve the architecture of islets with longer survival time and increased insulin content in STZ-induced diabetic mice. Histologic analysis has demonstrated that treatment with EVs improves the level of CD31 expression in pancreatic islets (which are markers of endothelial cells), indicating the enhanced islet vascularization (160). Interestingly, Cantaluppi et al. utilized EVs from endothelial progenitor cells with the islet transplantation mice model. Their study demonstrated that these EVs carry proangiogenic miR-126 and miR-196 enhanced islet vascularization, leading to sustained β-cell function (161). Nie et al. showed that human mesenchymal stem cell (MSC)-derived exosomes can improve the survival ratio, viability, and function of neonatal porcine islet cell clusters under hypoxic conditions (162), which are key factors causing islet graft dysfunction (163). Recently, Gesmundo et al. reported that adipocyte-derived EVs regulated the survival and function of human pancreatic β cells and pancreatic islets (164). For the role of EVs in islet transplantation, readers are encouraged to refer to the prior review (165). These findings support the idea that stem cell-derived EVs are suitable candidates to improve the functioning and survival of β cells for the treatment of T1D.

### Effects of Stem Cell-Derived EVs on Stem Cell Differentiation Toward β Cells

β-cell regeneration using stem cells means utilizing pluripotent stem cells with differentiation protocol to generate insulin-producing cells *in vitro*—a strategy for β-cell replacement therapy. Human pluripotent stem cells [either human embryonic stem cells (ESC) or induced pluripotent stem cells (iPS)] are attractive sources for β-cell differentiation since they can give rise to every cell type of the human body (166). To date, scientists have employed the multiple differentiation protocols, exposing cells to various growth factors and numerous signaling molecules in a particular sequence for differentiation of the cells into pancreatic endocrine cells (167, 168). Recently, other multipotent stem cells applied for the β-cell regeneration including MSCs and CB-SCs (169, 170). Interestingly, 3-dimensional cultures promote the differentiation of stem cells into insulin-producing cells with increased insulin and c-peptide secretion (171–173). Currently, there is no evidence suggesting that stem cell-derived EVs can definitively affect the differentiation of stem cells into functional islet β cells.

## Conclusions

Currently, most clinical trials on EVs or exosomes are considering their function as valuable biomarkers for diagnosis and prognosis in a range of diseases. Based on their capabilities of immune modulations and anti-inflammation, some of the studies are actively exploring EVs as therapeutic carriers by using MSC-derived EVs in cases of chronic kidney disease (174), lung injury (175), and severe COVID-19 (176, 177). To date, a number of preclinical studies have implied the translational capability of stem cell-derived EVs to treat T1D through their multiple immunomodulations of different immune cells and their potential to improve β-cell regeneration. That being said, only one clinical trial posted in ClinicalTrials.gov in 2014 (NCT02138331) did not report results. In comparison with their parent cells, stem cell-derived EVs may have good safety profiles and can be easily stored and transported as cell-free products without losing their functions. However, parent cells at different *ex vivo* culture conditions (e.g., culture medium with or without serum) may markedly affect their exosomes’ biochemical and biophysical features including the quantity and quality of bioactive molecules. Thus, it will be essential to develop a scalable and reproducible Standard Operating Procedure (SOP) for the EV production. Stem cell-derived EVs carry cargos of enriched biomolecules (RNAs, proteins) that need to be further characterized, clarifying their unique and synergistic effects for the treatment of T1D.

Overcoming the autoimmunity and shortage of islet β cells are two major issues for the treatment of T1D patients. Due to the limitations of native EVs such as the diversity, the low yield of EV production, as well as a short half-life and off-target effects of their actions post administration, it will be critical to direct a sufficient amount of EVs towards the specific targeting of autoimmune cells. Additionally, future attention should be placed on promoting the replication of residual β cells in pancreatic islets. To this respect, using the bioengineered EVs may facilitate the clinical translation of EVs for T1D treatment. However, the toxicity, purity, potency, and stability of these bioengineered EVs are mandatory for the FDA approval in clinical trials. These practical challenges must be overcome before stem cell-derived or bioengineered EVs can achieve their full therapeutic potentials for T1D and other autoimmune diseases.

## Author Contributions

YZ contributed to concepts, article revising, and final approval of article. WH and XS drafted the article. HY, JS, HW, and YZ edited the review article. All authors contributed to the article and approved the submitted version.

## Conflict of Interest

YZ is an inventor for Stem Cell Educator therapy and has a fiduciary role at Throne Biotechnologies.

The remaining authors declare that the research was conducted in the absence of any commercial or financial relationships that could be construed as a potential conflict of interest.

## Publisher’s Note

All claims expressed in this article are solely those of the authors and do not necessarily represent those of their affiliated organizations, or those of the publisher, the editors and the reviewers. Any product that may be evaluated in this article, or claim that may be made by its manufacturer, is not guaranteed or endorsed by the publisher.
